# Effect of trochanter displacement on impingement and bone contact in total hip arthroplasty performed after curved intertrochanteric varus osteotomy for osteonecrosis of the femoral head: a simulation study

**DOI:** 10.1186/s12891-022-05803-x

**Published:** 2022-09-08

**Authors:** Masanori Okamoto, Taisuke Seki, Yasuhiko Takegami, Yusuke Osawa, Shiro Imagama

**Affiliations:** Department of Orthopaedic Surgery, Nagoya University Graduate School of Medicine, 65 Tsurumai-cho, Showa-ku, Nagoya, Aichi 466-8550 Japan

**Keywords:** Curved intertrochanteric varus osteotomy, Osteonecrosis of the femoral head, Total hip arthroplasty, Computer simulation

## Abstract

**Background:**

Curved intertrochanteric varus osteotomy (CVO) is a useful treatment option for osteonecrosis of the femoral head (ONFH). However, the effect of proximal femoral deformity in cases of CVO converted to total hip arthroplasty (THA) remains unclear. The aim of this study was to evaluate the effect of trochanter displacement on impingement and the contact state of the implant and femur in THA.

**Methods:**

Thirty-eight hips that had undergone CVO for ONFH were reviewed and compared with a control group of 30 contralateral hips that had not undergone surgery. The range of motion (ROM) and impingement patterns and the percentage of cortical bone in the stem placement within the femur were measured by simulation using CT-based three-dimensional template software. We also measured the ROM and the number of joints that achieved the ROM required for activities of daily living when the upward displaced apex of the greater trochanter with osteotomy was resected and compared the findings with those obtained when the apex was not resected.

**Results:**

The CVO group showed a significantly greater bony impingement in external rotation (68.4% vs. 43.3%, *p* = 0.033) and abduction (78.9% vs. 33.3%, *p* < 0.001) than in the control group. The CVO group showed a significantly smaller range of external rotation (19.0° [interquartile range; 4.0–28.8] vs. 38.0° [interquartile range; 36.0–41.8], *p* < 0.001) and abduction (23.0° [interquartile range; 8.5–38.8] vs. 56.0° [interquartile range; 50.3–60.0], *p* < 0.001) than in the control group. Significantly more joints achieved the ROM necessary for activities of daily living when the apex was resected than when it was not (10.5% vs. 63.2%, *p* < 0.001). The percentage of cortical bone in the stem placement position was significantly higher in the CVO group than in the control group in the proximal portion of the stem (25.5% vs. 0.0%, *p* < 0.001).

**Conclusion:**

In cases requiring conversion to THA, we recommend resecting the upward displaced apex to achieve a sufficient ROM and carefully resecting the bone to avoid malignment of the stem.

## Introduction

Osteonecrosis of the femoral head (ONFH) occurs mostly in younger patients [1, 2]. In ONFH, the weight-bearing area progresses to collapse and eventually secondary osteoarthritis, restricting patients’ daily activities and consequently leading to reduced quality of life [3, 4]. Total hip arthroplasty (THA) is a reasonable option for advanced stages of the disease [5]. However, for young patients, the possibility of revisions remains a concern [6–8]. Thus, the patient's original joint should be maintained, if possible [9].

Several surgical treatments to preserve native joints have been described previously [10–16]. The indications for curved intertrochanteric varus osteotomy (CVO) offer the potential to cover more than one-third of the intact articular surface on the preoperative anteroposterior hip radiographs obtained at maximal abduction, resulting in an atraumatic rate of 30%. CVO is a useful treatment option for osteonecrosis of the femoral head ONFH. In ONFH cases where the indications for CVO are strictly adhered to, the patients’ functional outcomes, sports activity, and satisfaction level can be comparable to those achieved with THA. Previous studies have reported success rates of 90%–92% for CVO [15–17]. However, some patients with failed CVO have to undergo conversion to THA as a salvage operation. After CVO, the greater trochanter is elevated by 0.9–1.2 cm [15, 18].

Deformities in the proximal femur can have adverse effects the incidence of impingement and result in worsening of the cortical contact of the femoral implant in THA [19, 20]. However, few studies have evaluated these adverse effects. The purpose of this study was to clarify how the proximal femoral deformity introduced after CVO affects bony impingement and cortical contact of the femoral stem in cases requiring conversion to THA. We hypothesized that in THA after CVO, the apex of the greater trochanter displaced upward caused bony impingement, reducing the range of motion (ROM) and increasing cortical bone contact at the proximal portion of the stem due to deformation of the proximal femur.

## Material and methods

### Patients

This study was approved by the institutional review board of our institute. The aim of the study was explained to the patients, and written informed consent to participate in the study was obtained from them before they were included in the study. We retrospectively reviewed the findings for 116 hips of 106 patients who underwent CVO without acetabular osteotomy for the treatment of ONFH between August 1999 and March 2020. The following were excluded: 11 hips in 9 patients with cases already converted to THA, 57 hips in 62 patients not currently being followed up at our institution, and 5 hips in 5 patients for whom CT imaging was not performed. We included 38 hips in 35 patients in the analysis. The diagnosis of ONFH was based on clinical presentation and imaging studies, including plain radiography and magnetic resonance imaging [21]. All the operations were performed in accordance with the procedures described in previous reports [15]. The mean age at surgery was 34.8 ± 10.9 years, and the mean follow-up period was 114.5 ± 80.8 months. Among the contralateral hips of the same patients, eight hips in seven patients had previously undergone procedures such as osteotomy or THA. Thus, 30 hips from the remaining 28 patients that had not undergone any antecedent surgery were selected as the control group. Patient characteristics are shown in Table 1.

**Table 1:** Patient demographics

	CVO group (38 hips)	Control group (30 hips)	*p* value
Age at CVO (months)	34.5 [26.5–41.8]	35.5 [31.3–41.8]	0.688^a^
Age at simulation (months)	45.0 [35.3–52.8]	44.4 [38.0–52.3]	0.980^a^
Follow-up duration after CVO (months)	120.0 [27.5–192.8]	106.0 [12.0–188.5]	0.354^a^
Sex (female/male)	21/17	14/16	0.494^b^
Height (cm)	167.0 [156.3–170.8]	168.0 [157.3–171.0]	0.473^a^
Weight (kg)	61.8 [53.0–75.0]	65.5 [53.3–75.8]	0.753^a^
BMI (kg/m^2^)	22.7 [20.2–26.9]	22.7 [19.6–26.4]	0.970^a^

Values are presented as medians [interquartile range]

*CVO* Curved intertrochanteric varus osteotomy, *BMI* Body mass index

^a^Mann–Whitney U test

^b^Fisher's exact test

### Computer simulation

The patients underwent a preoperative CT examination that was performed from the anterior superior iliac spine to the knee joint through the distal femoral condyles by using a 320-row multidetector helical CT scanner (Aquilion One; Toshiba Medical Systems Co., Tochigi, Japan) with a 1.0-mm reconstructed slice. After downloading the scan data in the Digital Imaging and Communications in Medicine format (DICOM; NEMA [National Electrical Manufacturers Association], Rosslyn, VA, USA), computer simulations were performed using the CT-based simulation software ZedHip (Lexi Co. Ltd., Tokyo, Japan). This software was used to create virtual 3D bone models and perform preoperative THA planning and to simulate the ROM before impingement occurred between the implants and bones.

The implants used for the simulation were a G7 PPS BoneMaster Limited Hole cup, E1 acetabular liner, a Wagner Cone 135°, and a 32-mm ceramic head (Zimmer-Biomet, Warsaw, IN, USA). The stem was selected to match the deformity of the proximal femur [22]. We defined the pelvic coordinate system as relative to the functional pelvic plane. The acetabular component size was also chosen to maximize both fit and fill within the acetabulum, and the acetabular component was positioned at the site of the original acetabulum. The cup was positioned at an inclination and anteversion of 40° and 20°, respectively. The femoral-implant size was chosen to maximize both fit and fill in the femoral diaphysis to be fixed at the distal part of the stem. The femoral component axis was placed at the center of the femoral diaphysis, and the anteversion was set at 20° to the posterior condylar axis.

### ROM and impingement

The ROM was simulated and measured using three-dimensional template software. The pelvic coordinate system was used for the functional pelvic plane, while the femoral coordinate system was defined according to the guidelines of the International Society of Biomechanics [23]. ROM was measured during flexion (Flex), internal rotation (IR) at 90° flexion, external rotation (ER), and abduction (Ab) at the implant or the bony impingement. On the basis of the findings of previous studies, the following ROM conditions were defined as ROM values required for activities of daily living: Flex ≥ 110°, IR ≥ 30°, ER ≥ 30°, and Ab ≥ 0° [24, 25].

### Radiographic evaluation of femur morphology

**Fig. 1:**
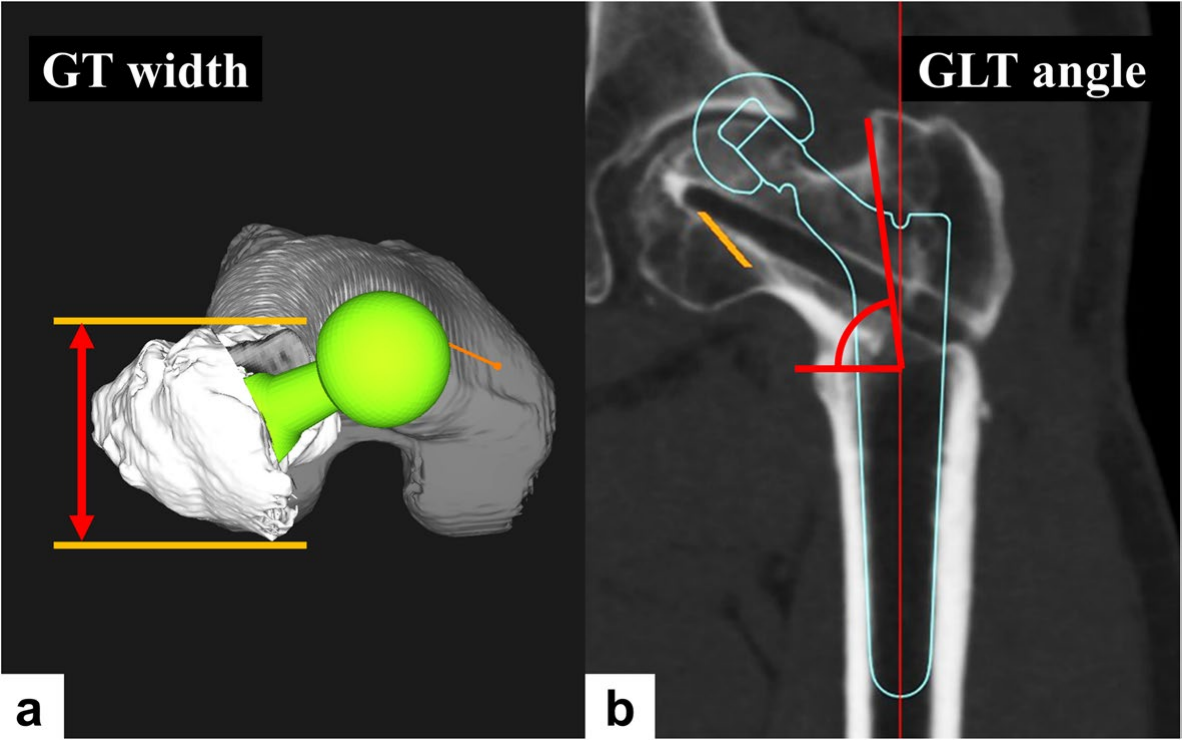
Setting greater trochanter width and greater and lesser trochanter angles. **a** The figure shows the axial plane of 3D model of the femur. The two-headed arrows refer to the vertical distance between the anterior and posterior femoral greater trochanter projected in the axial plane. **b** In the coronal plane of the femur, the angle between the greater and lesser trochanter at the level of the lesser trochanter represents the greater and lesser trochanter (GLT) angle. This angle decreases as the apex of the greater trochanter moves medially

The greater trochanter (GT) width [26], which is the vertical distance between the anterior and posterior femoral greater trochanter projected in the axial plane, was measured using three-dimensional templating software (Fig. 1a). The angle formed by the top of the greater trochanter and the lesser trochanter with the femoral shaft center at the height of the lesser trochanter as the vertex was measured in the coronal plane in the femoral coordinate system, and was defined as the greater and lesser trochanter (GLT) angle (Fig. 1b).

### Simulation of deformed greater trochanter resection

**Fig. 2:**
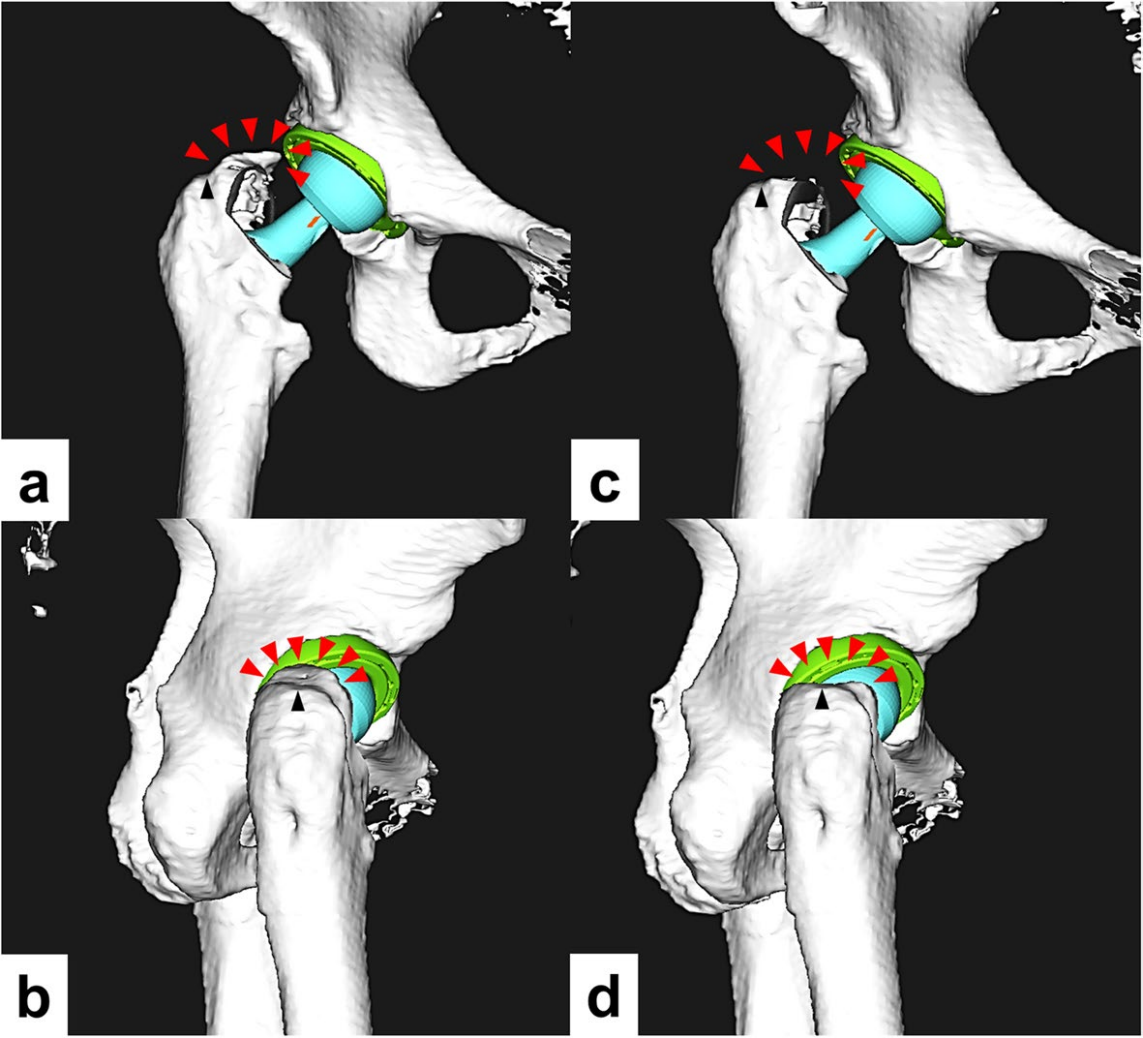
Three-dimensional model simulating total hip arthroplasty after curved intertrochanteric varus osteotomy. **a**, **b** The greater trochanter is deviated upward after the osteotomy. The red arrows indicate the deviated greater trochanter. The black arrows indicate the notch between the femoral shaft and the bone fragment that had moved varus. **c**, **d** To simulate the resection of the deviated greater trochanter, a three-dimensional model is generated with the greater trochanter resected all bone proximal to the notch

The effect of resection of the deformed greater trochanter after conversion to THA on the ROM was examined. The ROM required for activities of daily living in the CVO group was compared with the values in a model in which the greater trochanter was resected. Using the same software, a three-dimensional model in the CVO group was reconstructed in which the proximal portion of the greater trochanter was resected above the lateral notch between the femoral shaft and the bone fragment that had moved varus (Fig. 2). The implants were placed at the same position.

### Analyses of the contact area between the implant stem and femur and the resection volume of cortical bone

**Fig. 3:**
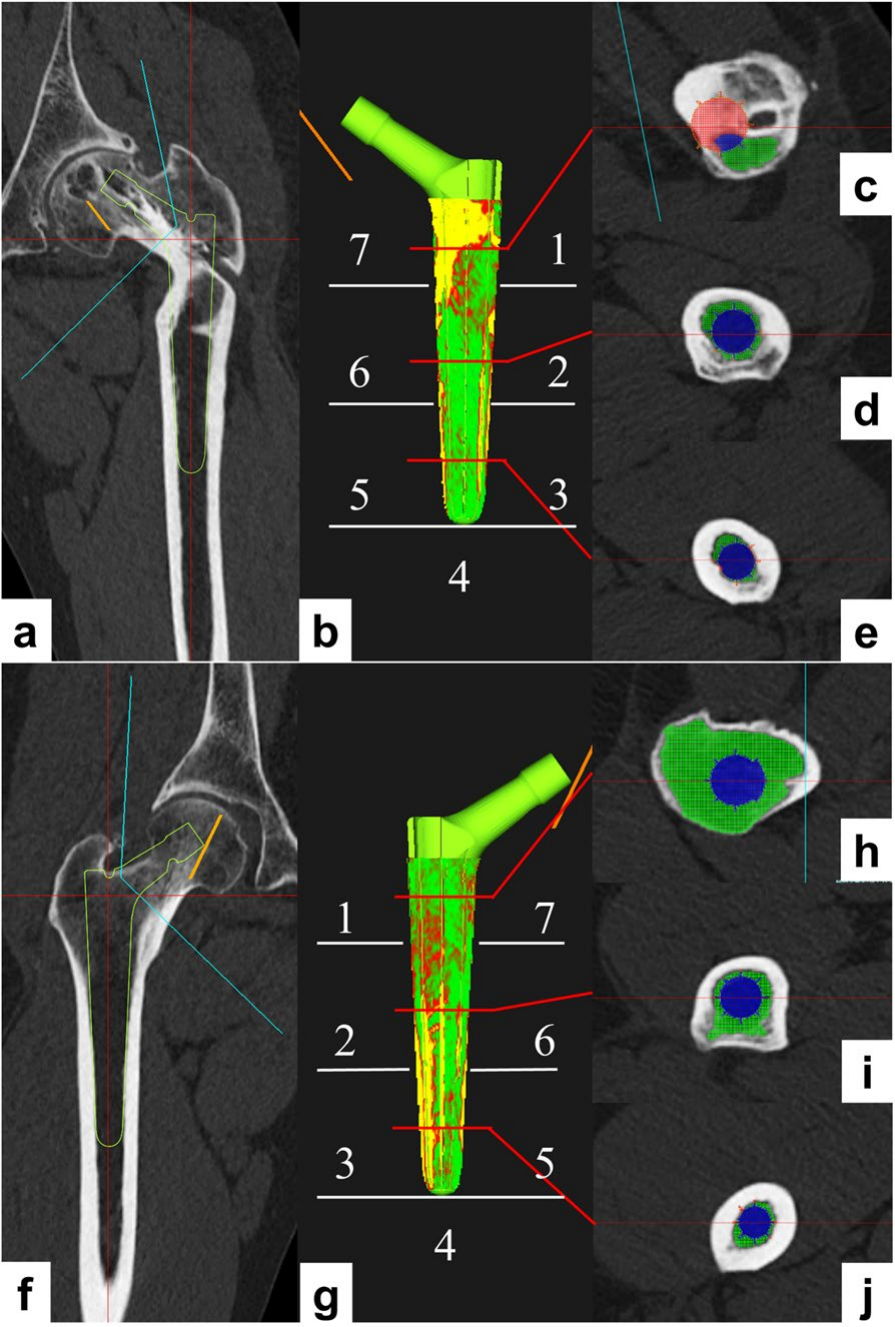
**a**-**e** This is an image of a curved intertrochanteric varus osteotomy (CVO) case. **f**-**j** This is an image of a control case. **a**, **f** Femoral implantation in a coronal view. **b**, **g** Density mapping shows the type of bone in contact with the implant by color. Yellow areas indicate contact with cortical bone, red with dense trabecular bone, and green with sparse trabecular bone. The numbers indicate the zone number of Gruen's zone classification. **c**, **h** Femoral implantation at the Gruen's zone 1, 7 in an axial view. The blue color indicates the stem in the intramedullary area. The red color indicates the stem in extramedullary area, in which cortical bone. The green color indicates the intramedullary area without the stem. The CVO case has many extramedullary areas. **d**, **h** Femoral implantation at the Gruen's zone 2, 6 in an axial view. **e**, **j** Femoral implantation at the Gruen's zone 3, 5 in an axial view

Using the same software, the mapping of the implant and femoral bone condition was visualized [26–28]. The analysis was performed in cases where the fixation instruments were removed to assess contact with the bone. The threshold of the cortical and cancerous bone interface was set to 543 HU in accordance with previous studies [26–28]. The percentage of the implant surface in contact with the cortical bone was measured. In addition, the percentages were measured for each Gruen’s zone classification [29] (Fig. 3). The software can measure the medullary cavity occupancy of the stem and the intramedullary and extramedullary volumes of the stem (Fig. 3). The percentage of extramedullary volume in the stem volume was measured. The extramedullary volume of the stem represents the sum of the volume of the cortical bone that was rasped or resected during stem insertion and the volume of the femoral defect. Then, according to Gruen’s zone classification, the proximal one-third of the stem was evaluated as “zone 1, 7”; the central one-third was evaluated as “zone 2, 6”; and the distal one-third was evaluated as “zone 3, 5”.

### Statistical analysis

The Mann–Whitney U test or Fisher’s exact test was used to compare the two groups. Correlations were evaluated using Pearson's chi-square test. Data were expressed as medians [interquartile range]. Statistical significance was set at *p* < 0.05. All statistical analyses were performed using the SPSS ver. 28 (IBM Corp. Armonk, NY, USA).

## Results

The CVO and control groups showed no significant differences in patient background factors (Table 1). For the analysis of the contact area between the implant stem and femur and the resection volume of cortical bone, 29 hips in the CVO group and 30 hips in the control group were included. The stem size was a median of 17.0 mm [16.0–18.8 mm] in the CVO group and 17.0 mm [16.0–19.8 mm] in the control group. Of the 38 hips in the CVO group, 17 hips were the same size as the contralateral hips, which were the control group. Ten hips were larger than the contralateral hips, three hips were smaller than the contralateral hips, and eight hips were not measured in the contralateral hip because of a history of surgery on the contralateral side.

The ROMs for ER and Ab in the CVO group were significantly smaller than those in the control group (19.0° [4.0°-28.8°] vs. 38.0° [36.0°-41.8°], *P* < 0.001; 23.0° [8.5°-38.8°] vs. 56.0° [50.3°-60.0°], *p* < 0.001) (Table 2). The number of cases of bony impingement in the ER and Ab in the CVO group was higher than that in the control group. No significant intergroup differences were observed for Flex and IR. The GLT angle was smaller in the CVO group than in the control group, although the GT width was not significantly different between the groups. A significant positive correlation was observed between the GLT angle and the ROM of the ER and Ab in the CVO group. There were no significant differences between GT width and the ROM (Table 3).

**Table 2:** Range of motion, type of impingement, and radiographic characteristics

	CVO group (38 hips)	Control group (30 hips)	*p* value
Range of motion (degree)			
Flex	119.0 [113.3–124.8]	114.0 [110.3–121.0]]	0.238^a^
IR	33.5 [27.3–41.0]	35.0 [29.5–39.8]	0.617^a^
ER	19.0 [4.0–28.8]	38.0 [36.0–41.8]	< 0.001^a^
Ab	23.0 [8.5–38.8]	56.0 [50.3–60.0]	< 0.001^a^
Impingement type (n, bony/implant)			
Flex	38/0	30/0	-
IR	31/7	29/1	0.058^b^
ER	26/12	13/17	0.033^b^
Ab	30/8	10/20	< 0.001^b^
GT width (cm)	42.9 [38.8–46.4]	40.2 [37.2–44.8]	0.137^a^
GLT angle (degrees)	83.2 [79.6–86.9]	93.4 [88.2–95.8]	< 0.001^a^

Values are presented as medians [interquartile range]

*CVO* Curved intertrochanteric varus osteotomy, *Flex* Flexion, *IR* Internal rotation, *ER* External rotation, *b* abduction, *GT* Width, greater trochanter width, *GLT* Angle, greater and lesser trochanter angle

^a^Mann–Whitney U test

^b^Fisher's exact test

**Table 3:** Relationship between radiographic characteristics and range of motion in the CVO group

	Flex		IR		ER		Ab	
	r	*p* value^a^	r	*p* value^a^	r	*p* value^a^	r	*p* value^a^
GT width	-0.214	0.198	-0.176	0.292	-0.262	0.112	-0.181	0.276
GLT angle	-0.216	0.193	0.036	0.830	0.698	< 0.001	0.648	< 0.001

*Flex* Flexion, *IR* Internal rotation, *ER* External rotation, *Ab* Abduction, *GT* Width, greater trochanter width, *GLT* angle, greater and lesser trochanter angle

^a^Spearman product-moment correlation coefficient

The results of a simulation in which the deviated greater trochanter was resected are shown in Table 4. The ROM in the ER and Ab in the group of patients with resection of the top of the greater trochanter increased significantly compared to the group with non-resection (36.0° [30.5°-39.0°] vs. 19.0° [4.0°-28.8°], *P* < 0.001; 49.5° [40.0°-57.8°] vs. 23.0° [8.5°-38.8°], *p* < 0.001) (Table 4). No significant differences were observed in Flex and IR. In the resection group, the number of cases of bony impingement in the Ab group was significantly lower than that in the non-resection group. In IR, ER, and Ab, the number of hips that achieved the required ROM for activities of daily living in the resection group was higher (Table 4).

**Table 4:** Range of motion, type of impingement, and number of hips achieving the required ROM for daily activities in the group that did not undergo resection of the top of the greater trochanter group and the group that did

	Non-resection of the top of greater trochanter (38 hips)	Resection of the top of greater trochanter (38 hips)	*p* value
ROM (degree)			
Flex	119.0 [113.3–124.8]	119.5 [114.3–125.8]	0.716^a^
IR	33.5 [27.3–41.0]	39.0 [30.3–43.8]	0.115^a^
ER	19.0 [4.0–28.8]	36.0 [30.5–39.0]	< 0.001^a^
Ab	23.0 [8.5–38.8]	49.5 [40.0–57.8]	< 0.001^a^
Impingement type (n, bony/implant)			
Flex	38/0	38/0	-
IR	31/7	32/6	0.050^b^
ER	26/12	21/17	0.123^b^
Ab	30/8	22/16	0.042^b^
Number of patients achieving ROM required for activities of daily living / not)			
Flex	32/6	33/5	0.500^b^
IR	21/17	31/7	0.013^b^
ER	9/29	31/7	< 0.001^b^
Ab	15/23	37/1	< 0.001^b^
All motions	4/34	24//14	< 0.001^b^

Values are presented as medians [interquartile range]

*Flex* Flexion, *IR* Internal rotation, *ER* External rotation, *Ab* Abduction, *ROM* Range of motion

^a^Mann–Whitney U test

^b^Fisher's exact test

The percentages of the contact area between the implant and femur in Gruen’s zones 1 and 7, which formed the proximal part of the stem, and zone 6 in the CVO group were significantly higher than those in the control group (Table 5). On the other hand, there was no difference in the percentages of the contact areas in Gruen’s zones 3 and 5, which formed the distal part of the stem, and zone 6. The percentage of extramedullary volume in the stem volume of Gruen’s zone 1 and 7 in CVO group was significantly greater than those in the control group (25.5% [7.7%-38.4%] vs. 0.0% [0.0%-0.2%], *P* < 0.001) (Table 6). Of the 29 hips, 4 had partial femoral bone defects around the stem in zone 7.

**Table 5:** The percentages of femoral-implant surface contact area for each Gruen’s zone

	CVO (29 hips)	Control (30 hips)	*p* value ^a^
Zone 1	8.2 [2.3–15.1]	0.3 [0.1–0.7]	< 0.001
Zone 2	2.2 [0.3–3.7]	4.1 [2.0–7.3]	0.038
Zone 3	1.8 [0.3–3.7]	2.2 [1.4–6.8]	0.139
Zone 5	4.6 [1.4–8.7]	3.8 [1.6–7.3]	0.471
Zone 6	2.1 [0.3–7.8]	1.6 [0.7–5.4]	0.844
Zone 7	26.2 [20.5–42.9]	2.0 [1.3–4.0]	< 0.001
Zone 1–3, 5–7	8.5 [5.2–12.8]	2.9 [1.6–5.1]	< 0.001

Values are presented as medians [interquartile range]

*CVO* Curved intertrochanteric varus osteotomy

^a^Mann–Whitney U test

**Table 6:** The Percentage of extramedullary volume to the stem volume for each trisection in the axial direction of the stem

	CVO (29 hips)	Control (30 hips)	*p* value ^a^
Zones 1, 7	25.5 [7.7–38.4]	0.0 [0.0–0.2]	< 0.001
Zones 2, 6	0.3 [0.1–0.9]	0.5 [0.1–0.9]	0.636
Zones 3, 5	0.7 [0.3–1.2]	0.8 [0.4–1.4]	0.301
Zones 1–3, 5–7	11.2 [3.4–17.8]	0.5 [0.2–0.9]	< 0.001

Values are presented as mean (standard deviation)

Zones 1, 7 show the proximal third; zones 2, 6 show the central third; and zones 3, 5 show the distal third

*CVO* Curved intertrochanteric varus osteotomy

^a^Mann–Whitney U test

## Discussion

This is the first study in which computer simulations were used to visualize the problems associated with THA after CVO. THA after CVO involved greater bony impingement in abduction and ER than the naïve hip and resulted in smaller ROMs. The apex of the greater trochanter was transformed to varus. Resection of the deformed apex decreased bony impingement, increased ROM, and provided a greater range of motion for activities of daily living. The cortical bone contact at the proximal part of the implant was high.

A previous study reported that the functional outcomes and patient satisfaction after CVO were comparable to those after THA, but the ROM in abduction was smaller [30]. Previous reports have demonstrated that the ROM of the hip joint decreased in patients who required conversion THA after transtrochanteric rotational osteotomy (TRO) [31–33]. A simulation study of THA after TRO showed that an increase in the GT width resulted in an increase in bony impingement and a reduction in ROM during flexion [26]. In the present study, no increase was observed in the GT width in THA after CVO, and the impingement and ROMs during flexion and internal rotation were similar to those in the control group. However, ROMs during ER and abduction were lower in the cases involving THA after CVO. This study showed that the bone deformity after CVO, especially adduction deviation of the apex of the greater trochanter, caused a reduction in ROM due to bony impingement of ER and abduction. ​Because the GLT angle was positively correlated with the ROM in ER and abduction, resection of the apex of the displaced greater trochanter was considered necessary to avoid bony impingement. Furthermore, in simulations in which the apex was resected, the ROM increased and reached the ROM required for activities of daily living.

The stem was inserted 2.1° in a valgus position (range, -2° to 6°) in the THA performed after failed CVO in a previous study [19]. Another report showed that although none of the varus and valgus abnormalities exceeded 5°, 75% of the stems were inserted in a position more valgus than 0° [20]. After CVO, significant new bone formation was observed in the medial intertrochanteric region. New sclerotic bone interferes with medial insertion of the stem. Takegami et al. recommended the use of burrs to remove sclerotic bone [19]. In the present study, the contact area of the cortical bone was larger in the proximal part of Gruen's zone, and the extramedullary volumes of the stem were larger, indicating the need to resect a large amount of sclerotic bone in the proximal part. Furthermore, in the proximal one-third, approximately one-fourth of the area where the stem should be inserted was filled with cortex. The results of this simulation study highlighted the difficulties associated with stem insertion and the need for careful planning by surgeons.

This study had several limitations. First, this was a single-center study, and these findings should be validated in multicenter studies. Moreover, the number of cases involving osteotomy of the femur was small. Second, because this was a CT-based simulation study, the effect of soft tissue was not considered. The ROM might have been overestimated. However, the ROM in THA after CVO remained inadequate for daily living because it should be smaller than the present results due to soft tissue impingement. Third, the effect on cortical bone contact depends on the shape of the implant. In THA performed after CVO, appropriate proximal fixation can be difficult due to deformation of the calcar, and we thought that a cone stem with distal fixation was appropriate. Fourth, the extramedullary volume included femoral bone defects, which may have been overestimated as the cortical bone volume to be resected. However, only four hips had defects, and these hips were highly deformed, which could make them more difficult to operate on. Finally, this study did not reveal the effect of resecting the deformed apex of the greater trochanter in a real case. In the future, the impact of osteotomy should be evaluated in real cases.

## Conclusion

Our study showed that a femoral deformity after CVO increases bony impingement and decreases the ROM for ER and abduction. In young patients with ONFH, CVO is the one of the surgical options. These findings highlight the need for preoperative planning to ensure better function in the unfortunate event that conversion to THA becomes necessary. We recommend resection of the upwardly displaced apex of the greater trochanter and careful resection of the new bone to avoid stem malalignment.

## Data Availability

The datasets used and/or analyzed during the current study are available from the corresponding author on reasonable request.

## Abbreviations

CVO: Curved intertrochanteric varus osteotomy
ONFH: Osteonecrosis of the femoral head
THA: Total hip arthroplasty
ROM: Range of motion
BMI: Body mass index
IR: Internal rotation
ER: External rotation
Ab: Abduction
